# 6-Hy­droxy-7,8-dimethyl­chroman-2-one

**DOI:** 10.1107/S1600536812029704

**Published:** 2012-07-04

**Authors:** Shailesh K. Goswami, Lyall R. Hanton, C. John McAdam, Stephen C. Moratti, Jim Simpson

**Affiliations:** a Department of Chemistry, University of Otago, PO Box 56, Dunedin, New Zealand

## Abstract

The title compound, C_11_H_12_O_3_, is essentially planar, with an r.m.s. deviation of 0.179 Å from the mean plane through the 14 non-H atoms in the mol­ecule. The benzene ring and the pyranone mean plane are inclined at 13.12 (6)° to one another and the pyran­one ring adopts a flattened chair conformation. In the crystal, O—H⋯O hydrogen bonds and C—H⋯O contacts form *R*_1_^2^(6) rings and link mol­ecules into chains along *b*. Additional C—H⋯O contacts generate inversion dimers, with *R*_2_^2^(8) ring motifs, and form sheets parallel to (-102) which are linked by C—H⋯π interactions.

## Related literature

For the synthesis, see: Lecea *et al.* (2010 ▶). For details of the Cambridge Structural Database, see: Allen (2002 ▶) and for related structures, see: Cameron *et al.* (2011 ▶); Goswami *et al.* (2011 ▶, 2012 ▶). For standard bond lengths, see: Allen *et al.* (1987 ▶) and for hydrogen-bond motifs, see: Bernstein *et al.* (1995 ▶).
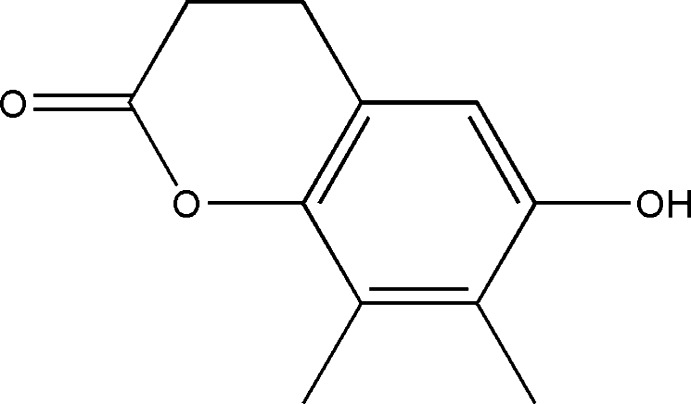


## Experimental

### 

#### Crystal data

C_11_H_12_O_3_
*M* *_r_* = 192.21
Triclinic, 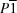

*a* = 6.2808 (14) Å
*b* = 8.630 (2) Å
*c* = 9.389 (2) Å
α = 88.603 (6)°
β = 83.638 (5)°
γ = 69.088 (5)°
*V* = 472.40 (19) Å^3^
*Z* = 2
Mo *K*α radiation
μ = 0.10 mm^−1^
*T* = 92 K
0.34 × 0.32 × 0.12 mm

#### Data collection

Bruker APEXII CCD area-detector diffractometer
Absorption correction: multi-scan (*SADABS*; Bruker, 2011 ▶) *T*_min_ = 0.656, *T*_max_ = 0.747
9073 measured reflections
3963 independent reflections
3368 reflections with *I* > 2σ(*I*)
*R*_int_ = 0.035

#### Refinement

*R*[*F* ^2^ > 2σ(*F* ^2^)] = 0.065
*wR*(*F* ^2^) = 0.188
*S* = 1.11
3963 reflections
132 parameters
H atoms treated by a mixture of independent and constrained refinement
Δρ_max_ = 0.60 e Å^−3^
Δρ_min_ = −0.28 e Å^−3^

### 

Data collection: *APEX2* (Bruker, 2011 ▶); cell refinement: *APEX2* (Bruker, 2011 ▶) and *SAINT* (Bruker, 2011 ▶); data reduction: *SAINT*; program(s) used to solve structure: *SHELXS97* (Sheldrick, 2008 ▶) and *TITAN2000* (Hunter & Simpson, 1999 ▶); program(s) used to refine structure: *SHELXL97* (Sheldrick, 2008 ▶) and *TITAN2000*; molecular graphics: *SHELXTL* (Sheldrick, 2008 ▶) and *Mercury* (Macrae *et al.*, 2008 ▶); software used to prepare material for publication: *SHELXL97*, *enCIFer* (Allen *et al.*, 2004 ▶), *PLATON* (Spek, 2009 ▶) and *publCIF* (Westrip, 2010 ▶).

## Supplementary Material

Crystal structure: contains datablock(s) global, I. DOI: 10.1107/S1600536812029704/lh5497sup1.cif

Structure factors: contains datablock(s) I. DOI: 10.1107/S1600536812029704/lh5497Isup2.hkl

Supplementary material file. DOI: 10.1107/S1600536812029704/lh5497Isup3.cml

Additional supplementary materials:  crystallographic information; 3D view; checkCIF report

## Figures and Tables

**Table 1:** Hydrogen-bond geometry (Å, °) *Cg* is the centroid of the C4–C9 benzene ring.

*D*—H⋯*A*	*D*—H	H⋯*A*	*D*⋯*A*	*D*—H⋯*A*
O8—H8*O*⋯O1^i^	0.89 (2)	1.89 (2)	2.7788 (15)	175 (2)
C9—H9⋯O1^i^	0.95	2.63	3.3371 (16)	132
C2—H2*A*⋯O1^ii^	0.99	2.52	3.4626 (16)	159
C3—H3*B*⋯*Cg* ^iii^	0.99	2.54	3.4771 (15)	157
C61—H61*C*⋯*Cg* ^iv^	0.98	2.79	3.6956 (16)	153

Symmetry codes: (i) 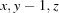; (ii) 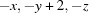; (iii) 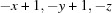; (iv) 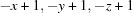.

**Fig. 1.**
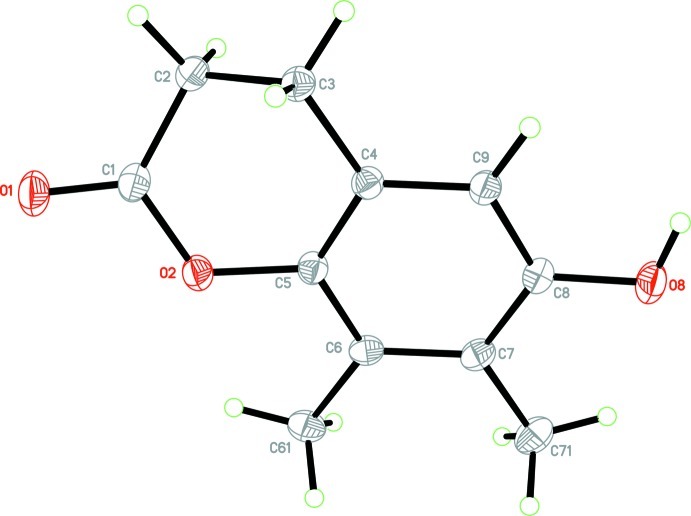
The structure of (I) with ellipsoids drawn at the 50% probability level.

**Fig. 2.**
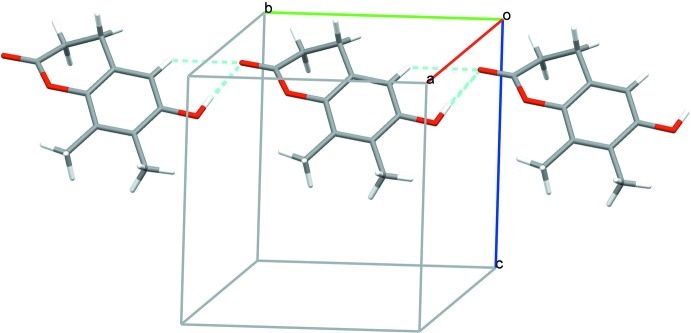
Rows of molecules along b linked by O—H···O and C—H···O hydrogen bonds drawn as dashed lines.

**Fig. 3.**
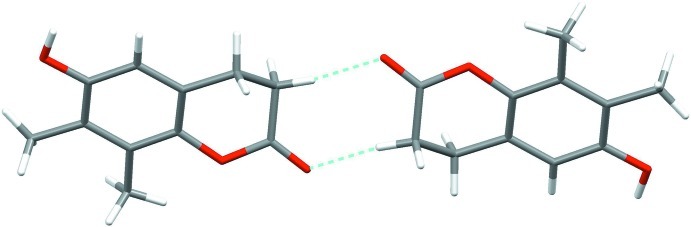
Inversion dimers formed by C—H···O hydrogen bonds drawn as dashed lines.

**Fig. 4.**
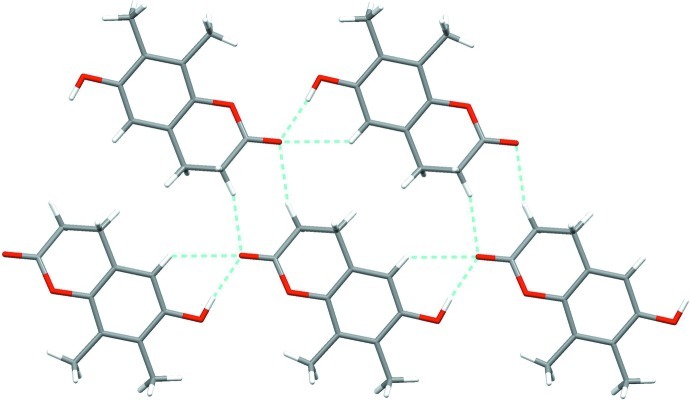
Sheets of molecules in the (-1,0,2) plane. Hydrogen bonds are drawn as dashed lines.

**Fig. 5.**
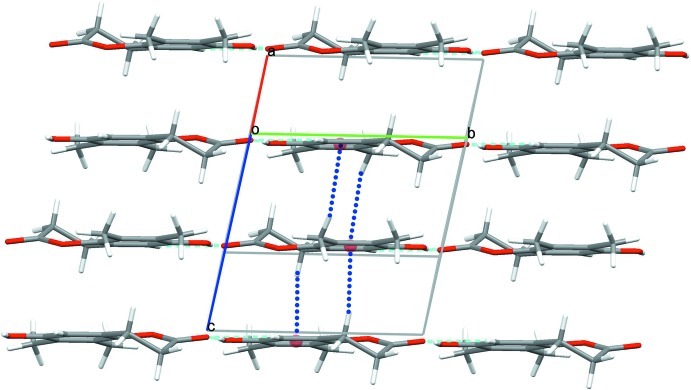
Overall packing of (I) showing representative C–H···π contacts as dotted lines. The red spheres represent the centroids of the C4···C9 benzene rings and hydrogen bonds are drawn as dashed lines.

## supplementary crystallographic information

### Comment

Our current research is focused on the preparation of quinone/hydroquinone based monomers for utilization in redox-active polymer gels. Synthesis of such systems is a multi-step process and often passes through a hydropyranone intermediate (Lecea *et al.*, 2010; Cameron *et al.*, 2011; Goswami *et al.*, 2011). The title compound illustrates one such intermediate and was isolated during the synthesis of a trifluoromethyl substituted hydroquinone.

The title compound (I), Fig 1, is almost planar with an r.m.s. deviation of 0.179 Å from the best fit plane through the 14 non-hydrogen atoms in the molecule. The maximum deviation from this plane is 0.5437 (11) Å for C2. This is in keeping with the fact that the pyranone ring adopts a flattened chair conformation with the C2 atom displaced by 0.6004 (17) Å from the plane through C1/O2/C5/C4/C3 which, in turn, has an r.m.s. deviation of 0.076 Å. This is in contrast to the closely related 5,6-dimethyl-1,2,9,10- tetrahydropyrano[3,2-*f*]chromene-3,8-dione (Goswami *et al.*, 2012), where both the C2 and O2 atoms of the pyranone rings were displaced significantly from the molecular plane in opposite directions. A search of the Cambridge Structural Database (Allen, 2002) revealed only two additional tetrahydropyrano derivatives (Goswami *et al.*, 2011, Cameron *et al.*, 2011). However, removing the restraint on substitution at the 3 and 4 positions of the pyranone ring, reveals the structures of more than 190 chromanone derivatives. The bond distances (Allen *et al.*, 1987) and angles in the molecule are normal and, despite the variation in the pyranone ring conformations, similar to those found in related structures (Goswami *et al.*, 2011, 2012; Cameron *et al.*, 2011).

In the crystal structure, O8—H8O···O1 hydrogen bonds, augmented by non-classical C9—H9···O1 contacts, form *R*^2^_1_(6) rings (Bernstein *et al.*, 1995) and link molecules into rows along *b*, Fig 2. C2—H2A···O1 hydrogen bonds form inversion dimers generating *R*^2^_2_(8) rings, Fig 3, which further connect the molecules into sheets approximately parallel to the (-1, 0, 2) plane, Fig 4. C—H···π contacts are also present linking adjacent molecules above and below the plane of the C4···C9 benzene ring and forming columns approximately orthogonal to the (-1, 0, 2) plane and resulting in a series of stacked layers, Fig 5.

### Experimental

The title compound was prepared according to the literature (Lecea *et al*., 2010) by a Friedel-Crafts type reaction of 2,3-dimethylhydroquinone with acrylic acid. X-ray quality crystals of (I) were grown from CDCl_3_.

### Refinement

Crystals of this material were not of good quality and the results presented here represent the best of several data collections. All H-atoms bound to carbon were refined using a riding model with d(C—H) = 0.99 Å, *U*_iso_ = 1.2*U*_eq_ (C) for methylene and 0.98 Å, *U*_iso_ = 1.5*U*_eq_ (C) for CH_3_ H atoms. The H8O hydrogen atom was located in a difference Fourier synthesis and its coordinates refined with *U*_iso_ = 1.5*U*_eq_ (O).

### Crystal data

**Table:**

C_11_H_12_O_3_	*Z* = 2
*M**_r_* = 192.21	*F*(000) = 204
Triclinic, *P*1	*D*_x_ = 1.351 Mg m^−^^3^
Hall symbol: -P 1	Mo *K*α radiation, λ = 0.71073 Å
*a* = 6.2808 (14) Å	Cell parameters from 4269 reflections
*b* = 8.630 (2) Å	θ = 2.5–35.1°
*c* = 9.389 (2) Å	µ = 0.10 mm^−^^1^
α = 88.603 (6)°	*T* = 92 K
β = 83.638 (5)°	Triangular plate, yellow
γ = 69.088 (5)°	0.34 × 0.32 × 0.12 mm
*V* = 472.40 (19) Å^3^	

### Data collection

**Table:**

Bruker APEXII CCD area-detector diffractometer	3963 independent reflections
Radiation source: fine-focus sealed tube	3368 reflections with *I* > 2σ(*I*)
Graphite monochromator	*R*_int_ = 0.035
φ and ω scans	θ_max_ = 35.1°, θ_min_ = 2.2°
Absorption correction: multi-scan (*SADABS*; Bruker, 2011)	*h* = −9→9
*T*_min_ = 0.656, *T*_max_ = 0.747	*k* = −13→12
9073 measured reflections	*l* = −14→15

### Refinement

**Table:**

Refinement on *F*^2^	Primary atom site location: structure-invariant direct methods
Least-squares matrix: full	Secondary atom site location: difference Fourier map
*R*[*F*^2^ > 2σ(*F*^2^)] = 0.065	Hydrogen site location: inferred from neighbouring sites
*wR*(*F*^2^) = 0.188	H atoms treated by a mixture of independent and constrained refinement
*S* = 1.11	*w* = 1/[σ^2^(*F*_o_^2^) + (0.0874*P*)^2^ + 0.1584*P*] where *P* = (*F*_o_^2^ + 2*F*_c_^2^)/3
3963 reflections	(Δ/σ)_max_ < 0.001
132 parameters	Δρ_max_ = 0.60 e Å^−^^3^
0 restraints	Δρ_min_ = −0.28 e Å^−^^3^

### Special details

**Table:**

Geometry. All e.s.d.'s (except the e.s.d. in the dihedral angle between two l.s. planes) are estimated using the full covariance matrix. The cell e.s.d.'s are taken into account individually in the estimation of e.s.d.'s in distances, angles and torsion angles; correlations between e.s.d.'s in cell parameters are only used when they are defined by crystal symmetry. An approximate (isotropic) treatment of cell e.s.d.'s is used for estimating e.s.d.'s involving l.s. planes.
Refinement. Refinement of *F*^2^ against ALL reflections. The weighted *R*-factor *wR* and goodness of fit *S* are based on *F*^2^, conventional *R*-factors *R* are based on *F*, with *F* set to zero for negative *F*^2^. The threshold expression of *F*^2^ > σ(*F*^2^) is used only for calculating *R*-factors(gt) *etc*. and is not relevant to the choice of reflections for refinement. *R*-factors based on *F*^2^ are statistically about twice as large as those based on *F*, and *R*- factors based on ALL data will be even larger.

### Fractional atomic coordinates and isotropic or equivalent isotropic displacement parameters (Å^2^)

**Table:**

	*x*	*y*	*z*	*U*_iso_*/*U*_eq_	
O1	0.26085 (18)	1.00639 (11)	0.13645 (11)	0.0271 (2)	
C1	0.2601 (2)	0.86709 (14)	0.15707 (13)	0.0195 (2)	
O2	0.44911 (14)	0.75378 (10)	0.20340 (9)	0.01890 (18)	
C2	0.0627 (2)	0.81355 (14)	0.13980 (13)	0.0198 (2)	
H2A	−0.0395	0.8915	0.0761	0.024*	
H2B	−0.0265	0.8178	0.2344	0.024*	
C3	0.14248 (19)	0.63811 (13)	0.07686 (12)	0.0175 (2)	
H3A	0.0114	0.5993	0.0819	0.021*	
H3B	0.2033	0.6378	−0.0251	0.021*	
C4	0.32642 (18)	0.52366 (13)	0.15984 (11)	0.01564 (19)	
C5	0.46469 (18)	0.58834 (13)	0.22549 (11)	0.01568 (19)	
C6	0.63239 (18)	0.49425 (14)	0.31075 (11)	0.0165 (2)	
C61	0.7683 (2)	0.57324 (16)	0.38576 (13)	0.0213 (2)	
H61A	0.7096	0.6933	0.3707	0.032*	
H61B	0.9300	0.5266	0.3468	0.032*	
H61C	0.7538	0.5514	0.4886	0.032*	
C7	0.66848 (19)	0.32489 (14)	0.32618 (12)	0.0179 (2)	
C71	0.8441 (2)	0.21682 (16)	0.41777 (14)	0.0247 (2)	
H71A	0.9982	0.1949	0.3685	0.037*	
H71B	0.8197	0.1116	0.4349	0.037*	
H71C	0.8290	0.2735	0.5096	0.037*	
C8	0.53406 (19)	0.25683 (13)	0.25696 (12)	0.0181 (2)	
O8	0.57401 (17)	0.09172 (11)	0.27400 (11)	0.0255 (2)	
H8O	0.472 (4)	0.070 (3)	0.226 (2)	0.038*	
C9	0.36335 (19)	0.35568 (13)	0.17644 (12)	0.0175 (2)	
H9	0.2715	0.3080	0.1326	0.021*	

### Atomic displacement parameters (Å^2^)

**Table:**

	*U*^11^	*U*^22^	*U*^33^	*U*^12^	*U*^13^	*U*^23^
O1	0.0341 (5)	0.0165 (4)	0.0344 (5)	−0.0117 (3)	−0.0113 (4)	0.0055 (3)
C1	0.0231 (5)	0.0153 (4)	0.0197 (5)	−0.0058 (4)	−0.0048 (4)	0.0014 (3)
O2	0.0213 (4)	0.0159 (4)	0.0221 (4)	−0.0087 (3)	−0.0064 (3)	0.0024 (3)
C2	0.0190 (5)	0.0157 (4)	0.0241 (5)	−0.0046 (4)	−0.0059 (4)	0.0015 (4)
C3	0.0189 (5)	0.0166 (4)	0.0176 (4)	−0.0060 (4)	−0.0059 (4)	0.0012 (3)
C4	0.0162 (4)	0.0146 (4)	0.0159 (4)	−0.0048 (3)	−0.0028 (3)	−0.0008 (3)
C5	0.0172 (4)	0.0147 (4)	0.0154 (4)	−0.0058 (3)	−0.0028 (3)	0.0008 (3)
C6	0.0148 (4)	0.0193 (5)	0.0147 (4)	−0.0052 (3)	−0.0021 (3)	−0.0003 (3)
C61	0.0191 (5)	0.0271 (6)	0.0200 (5)	−0.0102 (4)	−0.0050 (4)	0.0002 (4)
C7	0.0165 (4)	0.0189 (5)	0.0162 (4)	−0.0036 (4)	−0.0034 (3)	0.0020 (3)
C71	0.0237 (5)	0.0241 (5)	0.0228 (5)	−0.0027 (4)	−0.0087 (4)	0.0046 (4)
C8	0.0191 (5)	0.0145 (4)	0.0194 (5)	−0.0041 (3)	−0.0026 (4)	0.0011 (3)
O8	0.0281 (5)	0.0139 (4)	0.0342 (5)	−0.0052 (3)	−0.0101 (4)	0.0039 (3)
C9	0.0177 (5)	0.0146 (4)	0.0197 (5)	−0.0048 (3)	−0.0038 (4)	−0.0001 (3)

### Geometric parameters (Å, º)

**Table:**

O1—C1	1.2145 (14)	C6—C61	1.5039 (16)
C1—O2	1.3489 (14)	C61—H61A	0.9800
C1—C2	1.4948 (17)	C61—H61B	0.9800
O2—C5	1.4076 (13)	C61—H61C	0.9800
C2—C3	1.5261 (16)	C7—C8	1.4051 (16)
C2—H2A	0.9900	C7—C71	1.5044 (16)
C2—H2B	0.9900	C71—H71A	0.9800
C3—C4	1.5049 (15)	C71—H71B	0.9800
C3—H3A	0.9900	C71—H71C	0.9800
C3—H3B	0.9900	C8—O8	1.3644 (14)
C4—C5	1.3882 (15)	C8—C9	1.3943 (15)
C4—C9	1.3916 (15)	O8—O1^i^	2.7788 (15)
C5—C6	1.3979 (15)	O8—H8O	0.89 (2)
C6—C7	1.4032 (16)	C9—H9	0.9500
			
O1—C1—O2	117.42 (11)	C7—C6—C61	120.86 (10)
O1—C1—C2	124.89 (11)	C6—C61—H61A	109.5
O2—C1—C2	117.65 (10)	C6—C61—H61B	109.5
C1—O2—C5	120.91 (9)	H61A—C61—H61B	109.5
C1—C2—C3	111.78 (9)	C6—C61—H61C	109.5
C1—C2—H2A	109.3	H61A—C61—H61C	109.5
C3—C2—H2A	109.3	H61B—C61—H61C	109.5
C1—C2—H2B	109.3	C6—C7—C8	119.04 (10)
C3—C2—H2B	109.3	C6—C7—C71	121.15 (10)
H2A—C2—H2B	107.9	C8—C7—C71	119.79 (10)
C4—C3—C2	109.49 (9)	C7—C71—H71A	109.5
C4—C3—H3A	109.8	C7—C71—H71B	109.5
C2—C3—H3A	109.8	H71A—C71—H71B	109.5
C4—C3—H3B	109.8	C7—C71—H71C	109.5
C2—C3—H3B	109.8	H71A—C71—H71C	109.5
H3A—C3—H3B	108.2	H71B—C71—H71C	109.5
C5—C4—C9	117.89 (10)	O8—C8—C9	121.51 (10)
C5—C4—C3	118.78 (9)	O8—C8—C7	117.50 (10)
C9—C4—C3	123.32 (10)	C9—C8—C7	120.98 (10)
C4—C5—C6	123.13 (10)	C8—O8—H8O	105.9 (13)
C4—C5—O2	120.75 (9)	C4—C9—C8	120.53 (10)
C6—C5—O2	116.05 (9)	C4—C9—H9	119.7
C5—C6—C7	118.36 (10)	C8—C9—H9	119.7
C5—C6—C61	120.77 (10)		

Symmetry code: (i) *x*, *y*−1, *z*.

### Hydrogen-bond geometry (Å, º)

Cg is the centroid of the C4–C9 benzene ring.

**Table:**

*D*—H···*A*	*D*—H	H···*A*	*D*···*A*	*D*—H···*A*
O8—H8*O*···O1^i^	0.89 (2)	1.89 (2)	2.7788 (15)	175 (2)
C9—H9···O1^i^	0.95	2.63	3.3371 (16)	132
C2—H2*A*···O1^ii^	0.99	2.52	3.4626 (16)	159
C3—H3*B*···*Cg*^iii^	0.99	2.54	3.4771 (15)	157
C61—H61*C*···*Cg*^iv^	0.98	2.79	3.6956 (16)	153

Symmetry codes: (i) *x*, *y*−1, *z*; (ii) −*x*, −*y*+2, −*z*; (iii) −*x*+1, −*y*+1, −*z*; (iv) −*x*+1, −*y*+1, −*z*+1.
